# Soil organic matter dynamics in semiarid agroecosystems transitioning to dryland

**DOI:** 10.7717/peerj.10199

**Published:** 2020-10-20

**Authors:** Rajan Ghimire, Babu Ram Khanal

**Affiliations:** 1 Department of Plant and Environmental Sciences, New Mexico State University, Las Cruces, NM, United States of America; 2 Agricultural Science Center, New Mexico State University, Clovis, NM, United States of America; 3 Department of Soil Science and Agricultural Engineering, Agriculture and Forestry University, Rampur, Nepal

**Keywords:** Soil organic carbon, Nutrient cycling, Ogallala aquifer, Semiarid soils

## Abstract

Recent interest in improving soil health and agricultural sustainability recognizes the value of soil organic carbon (SOC) sequestration and nutrient cycling. The main goal of this study was to evaluate the response of various SOC and nitrogen (N) components in semiarid cropping systems transitioning from limited-irrigation to dryland and a restored grassland in the Southern High Plains of USA. Cropping systems evaluated include dryland winter wheat (*Triticum aestivum* L.)–sorghum (*Sorghum bicolor* L.)–fallow with conventional tillage (DLCTF) and no-tillage (DLNTF), limited-irrigation winter wheat–sorghum–fallow with no-tillage and cover cropping (LINTC) and no-tillage fallow (LINTF), and an undisturbed grassland (NG). Soil samples were collected from 0–15 cm and 15–30 cm depths and analyzed for SOC, total N, inorganic N, and soil microbial biomass carbon (SMBC) contents. The CO_2_ and N_2_O release during a eight-weeks long laboratory incubation were also analyzed. Results show 14% and 13% reduction in SOC and total N from 0–30 cm depth with the transition from limited-irrigation to dryland cropping systems while 51% more SOC and 41% more total N with the transition to grassland. The SMBC was 42% less in dryland cropping systems and 100% more in NG than the limited-irrigation cropping systems. However, the grassland was N limited, with 93% less inorganic N in NG compared to only 11% less in dryland cropping systems than in limited-irrigation cropping systems. The microbial respiration measured as CO_2_-C was highest in NG, followed by limited-irrigation and dryland cropping systems. The N_2_O-N release showed the lowest rate of N loss from dryland cropping systems, followed by NG and limited-irrigation cropping systems. This study demonstrated loss of SOC and N in agroecosystems transitioned to dryland crop-fallow systems, with greater magnitude of change observed in the biologically active fraction of soil organic matter. Grassland restoration could be an important strategy to increase SOC and nutrients in hot, dry, semiarid agroecosystems transitioning to dryland.

## Introduction

The rapid expansion of dryland areas in recent years significantly depleted SOC and increased greenhouse gas (GHG) emissions (Huang et al., 2016). With projected changes in climate, global population, and land-use, it is anticipated that dryland will cover half the world’s land surface by the end of the century and directly impact the livelihoods of ∼250 million people through degradation of soil and environmental quality (Huang et al., 2016; Reynolds et al., 2007). The High Plains region of USA, where the Ogallala Aquifer underlies about 450,660 km^2^ of the area, produces 30% of total crop and animal products in the country (Guerrero et al., 2010; NASS, 2016). Agricultural intensification using irrigation water pumped from the Ogallala Aquifer has regulated SOC and nutrient cycling, crop yields, and the rural economy. Land production values have increased by more than $12 billion annually after irrigation systems were developed (Hornbeck & Keskin, 2014). However, the saturated thickness of the Ogallala Aquifer has declined by more than 50% since its pre-development state in the Southern High Plains (Haacker, Kendall & Hyndman, 2016), with projected increase in dryland area. The extent of dryland expansion and the associated economic impacts remains unknown due to a lack of spatially explicit data on the Aquifer depletion in the region (Deines et al., 2020). Improved knowledge of SOC and N cycling during dryland transition will help in the development of a farming strategy that sustains agriculture in the regions facing challenges due to water limitation.

Management practices that increase SOC storage and improve soil health have been increasingly promoted for maximizing the economic potential of the land while enhancing environmental quality. Soil disturbance associated with agriculture around the globe has led to a loss of ∼133 Gt of soil C as CO_2_ to the atmosphere in the past century and affected crop production and the environment (Sanderman, Hengl & Fiske, 2017). The depletion of SOC and N is associated with increased soil erosion and decreased water storage, nutrient cycling, and soil biological activity, ultimately leading to reduced crop yields and profitability (Baumhardt, Stewart & Sainju, 2015; Ghimire, Machado & Bista, 2018). In contrast, increased biomass C input through crop rotations along with conservation tillage systems, nutrients, and soil amendment additions can increase SOC sequestration and nutrient cycling (Al-Kaisi, Yin & Licht, 2005; Sainju, Jabro & Stevens, 2008). Soils with no-tillage and cover cropping store more SOC and nutrients than conventional tillage soils without a cover crop (Mitchell et al., 2015). No-tillage with cover cropping also served as a net sink for GHG (De Gryze et al., 2011). No-tillage and cover crops increase microbial abundance and diversity, which is often associated with increased SOC accumulation, reduced GHG emissions, and improved soil health (Mondal et al., 2020; Schmidt, Mitchell & Scow, 2019).

Grassland restoration could be another strategy to increase SOC storage in agroecosystems transitioning from irrigated to dryland management. A recent study documented up to a 37% increase in SOC with grassland restoration (∼50 years), which was not significantly different from SOC in a lightly grazed native pasture under dryland condition (Ghimire et al., 2019). The increase in SOC and nutrients with grassland restoration in the previously cultivated soils have also been documented from other semiarid regions of the world, the magnitude of increase varying with management practices and environment (Liu et al., 2017; Yang et al., 2019). Native grasses often have denser and deeper root systems than annual crops, and increased SOC with grassland restoration often results from the high root biomass of perennial species and addition of root-derived C (Roumet, Urcelay & Díaz, 2006).

Early response to management changes can be evaluated by monitoring labile SOC and N fractions, including CO_2_ and N_2_O emissions, because the labile organic matter changes rapidly with alternative management systems (Franzluebbers & Stuedemann, 2008). Although physical, chemical, and biological transformation processes convert plant residues into organic matter, the biologically active fraction of SOC could be affected by management changes more rapidly than other fractions (Lehmann & Kleber, 2015), while the soil disturbance and long-fallow periods can lead to the rapid loss of biologically active SOC and N fractions conservation systems that reduce tillage and increase cropping intensity and diversity can improve SOC storage and nutrient cycling (Ghimire, Machado & Bista, 2019). The conservation systems may be important for the Southern High Plains agroecosystems, where hot, dry environment and intermittent irrigation favors rapid mineralization of organic matter and limits biomass C inputs, as well as microbial activities (Cano et al., 2018; Ghimire et al., 2019). Improved understanding of management practices that affect various SOC and N fractions will help in designing sustainable cropping systems in semiarid regions in the time of uncertainty due to decreasing irrigation capacity.

This study aimed to evaluate SOC and N components, including CO_2_ and N_2_O emissions during laboratory incubation of soil samples, in irrigated and dryland cropping systems with varying crop diversity and tillage intensity, and a restored grassland. We hypothesized that grassland restoration and adoption of conservation systems that reduce soil disturbance along with cover cropping could improve soil health in hot, dry, semiarid environments through increased SOC and N storage.

## Materials & Methods

### Study site and treatments

Soil samples for this study were collected from five experimental plots at the New Mexico State University Agricultural Science Center (ASC) at Clovis, NM (34°35′N, 103°12′W, 1348 m elevation). The soils at the study site are Olton clay loam (*fine, mixed, superactive, thermic Aridic Paleustolls*) based on USDA soil taxonomy (Soil Survey Staff, 2018). All the plots had a <1% slope. Soil bulk density ranged from 1.06 to 1.3 g cm^−3^, pH 6.9 to 8.0, electrical conductivity from 0.11 to 0.49 ds m^−1^ at the 0- to 30-cm depth. This area receives an average annual rainfall of 466 mm and yearly minimum and maximum temperatures of 4.28 °C and 22.1 °C.

The study plots included dryland winter wheat–sorghum–fallow with conventional tillage (DLCTF) and no-tillage management (DLNTF), limited-irrigation winter wheat–sorghum–fallow with no-tillage alone (LINTF) and no-tillage with cover cropping (LINTC), and a nearby native grassland (NG) as a reference. The entire experimental area was occasionally flood-irrigated until 2003. With the establishment of a center-pivot irrigation system in 2004, LINTF and LINTC plots were maintained under center pivot irrigation, conventional tillage and winter wheat, corn (*Zea mays* L.), and sorghum in rotation until 2014. Starting fall 2015, no-tillage winter wheat-sorghum-fallow rotation was established in LINTF plots, and a mixture of oat (*Avena sativa* L.), barley (*Hordeum vulgare* L.), pea (*Pisum sativum* L.), hairy vetch (*Vicia villosa* L.), winter canola (*Brassica napus* L.), and forage radish (*Raphanus sativus* L.) was planted as cover crops during the fallow period after each wheat and sorghum crop in LINTC plots. The typical winter wheat–sorghum–fallow leaves 11 month fallow after wheat harvest and 10 month fallow after sorghum harvest. In LINTC, cover crops were sown in the last week of February and terminated in the second week of May (∼90 days) after each crop in the rotation. Because of the declining irrigation water availability in the Ogallala Aquifer region, winter wheat and sorghum planted in these pltos receive only ∼50% of the typical irrigation needed for the respective crops. DLCTF and DLNTF plots were maintained under conventional tillage dryland winter wheat–sorghum–fallow rotation from 2003 to 2014. All plots used three to five tillage passes of a disk, cultivator, DMI Ripper, and land finisher to the depth of 0.15 m before planting wheat and sorghum. In 2015, DLNTF plots were converted to no-tillage management and maintained in the same practice since. Winter wheat is typically sown in the second week of October and sorghum is sown in the first week of June. Sorghum was planted in all plots during 2018, the year the soil samples were collected. The NG plots were not cultivated since 2004 and predominantly covered with plains lovegrass (*Eragrostis intermedia* Hitchc) and blue grama (*Bouteloua gracilis*).

The dryland and limited-irrigation cropland plots were part of a randomized experiment with three replications with a plot size of 18 m ×12 m for each treatment while NG plots were established in large fields (>1 ha area) with three randomly selected 6 m ×6 m plots at least 50 m apart from each other. Soil fertility management for each cropland plot was based on soil test recommendations. For example, sorghum received 97 kg N ha^−1^ and 15 kg S ha^−1^ under limited-irrigation systems and 34 kg N ha^−1^ and 4 kg S ha^−1^ under dryland systems in 2018. A mixture of urea, ammonium thiosulfate, and ammonium nitrate were applied at the time of planting. Winter wheat in 2017 received 70 kg N ha^−1^ and 12.5 kg S ha^−1^ under limited irrigation and 34 kg N ha^−1^ and 4 kg S ha^−1^ under dryland. Native grassland plots were not fertilized, nor the grasses were harvested or grazed.

### Soil sampling and laboratory analysis

Soil samples were collected from the 0–15 cm and 15–30 cm depths of five randomly selected spots within each plot after sorghum harvest in fall 2018 using a two cm inner diameter core sampler. The samples were composited by depth, and approximately 400-g samples free of roots, leaves, and other foreign materials were brought to the laboratory. All the soil samples were stored at 4 °C in the refrigerator until they were analyzed for labile SOC and N components, which was done within a week of soil sample collection. The remaining soils were air-dried, ground to pass through a 2-mm sieve for the analysis of other soil properties.

In the laboratory, inorganic N was analyzed by extracting 5-g soil samples in 25 mL of 1 mole L^−1^KCl and subsequently quantifying nitrate (NO_3_^−^) and ammonium (NH_4_^+^) ions in the supernatant using a Timberline N analyzer (Timberline Instruments, LLC, Boulder, CO). The SMBC was measured by the fumigation incubation method in which 10-g soil samples were brought to field capacity, placed in a vacuum desiccator, and fumigated for 48 hr using chloroform, followed by aerobic incubation in 1 L glass jars for a week. The CO_2_-C released from each setup during incubation was measured in an LI-820 (LI-COR Biosciences, Lincoln, NE). The SMBC was calculated by dividing the CO_2_-C released during incubation with 0.41 without subtracting non-fumigated control (Franzluebbers & Stuedemann, 2008). Soil pH and electrical conductivity (EC) were measured in 1:5 soil to water suspension using electrodes. Cation exchange capacity (CEC) was determined by extracting soil in 1N ammonium acetate (NH4OAc) solution followed by quantifying cations in an Inductively Coupled Plasma Spectrometer. Soil bulk density was determined by using the core method and SOC and total N was determined in a dry combustion analyzer (LECO Corporation, St. Joseph, MI, USA). Soils were high in inorganic C. Therefore, the soil samples were treated with a 6 mole L^−1^HCl solution before SOC and N analysis. Soil bulk density was used to convert SOC and N from per unit mass to volume of soil and the SOC and N stocks in 0–30 cm soil were calculated by summing the respective fractions in 0–15 cm and 15–30 cm depths.

Homogenized and composited soils from the 0–15 cm depth of each plot were used for the laboratory CO_2_ and N_2_O emissions study—the laboratory study used approximately 100 g soil samples in a 500 mL glass jar. Soil samples were incubated in a dark cabinet for 60 days at room temperature (23 ± 2 °C). The glass jars had an aluminum lid with a rubber septum for gas extractions. The CO_2_ and N_2_O gas produced during incubation was measured with a portable EGM-5 CO_2_ analyzer (PP systems Inc. Amesbury, MA, USA) and MIRA PICO N_2_O analyzer (AERIS Technologies Inc. Redwood City, CA, USA) using method described by Thapa et al. (2019) for CO_2_ emissions with modifications to conform to the laboratory conditions. In brief, a syringe connected to the analyzers through a rubber tubing was inserted into a septum on the lid of each jar was connected to the CO_2_ analyzer and the gas-out port from the CO_2_ analyzer was connected to the N_2_O analyzer. The concentrations of CO_2_ and N_2_O were measured in 1, 2, 3, 4, 8, 12, and 14 days of incubation, and in a weekly interval afterward until 8 weeks. After each sampling, lids of incubation jars were open, and gas concentrations were re-equilibrated by flushing with a vacuum pump. The jars were weighed every week to estimate the water loss, and the soil water content was brought to the original level by adding distilled water.

### Statistical analysis and greenhouse gas emissions modeling

All the soil data were analyzed by using a MIXED model procedure of a statistical analysis system (SAS v.9.4, SAS Institute, Cary, NC). All the data were tested for and met the homogeneity of variance and normality of residuals criteria. All the soil data (pH, EC, CEC, bulk density [Db], SOC, TN, inorganic N, and SMBC) were analyzed considering treatments as fixed and replication as a random factor. Treatment means were compared at *p* ≤ 0.05 unless otherwise stated.

The CO_2_-C and N_2_O-N concentrations in each jar (*R*) were calculated using equation suggested in Salehin et al. (2020), and the hourly CO_2_-C and N_2_O-N fluxes were converted to daily emissions rate and linearly interpolated to calculate the cumulative flux of the respective gases for the period soils were incubated. The first-order kinetic model (Eq. (1)) was fitted with cumulative emissions data to calculate potential C and N loss from each system as CO_2_ and N_2_O (Broadbent, 1986).

(1) \begin{eqnarray*}{Y}_{m}=X\times {t}^{k}\ldots \ldots \ldots \ldots \ldots \ldots \ldots \ldots \ldots .\end{eqnarray*}

Where *Y*_*m*_ is modeled C or N loss as CO_2_-C and N_2_O-N, *X* is a labile substrate in the soil (mg kg^−1^soil), *t* is the decomposition time (days), and *k* is the decomposition rate constant (day^−1^). Model performance was tested using Pearson’s correlation coefficient (r) for observed and modeled values, root means square error (RMSE), and normalized root means square error (NRMSE). Higher *r* value and lower RMSE and NRMSE values indicated a good fit of the model. Since SOC and TN contents were not consistent among treatments, the CO_2_-C and N_2_O-N release data were divided by SOC and TN content, respectively, and reanalyzed to estimate the CO_2_-C and N_2_O-N emissions per unit SOC and TN contents, respectively. PROC REG procedure was used for regression analysis.

## Results

Soil pH and EC varied significantly between treatments in both 0–15 cm and 15–30 cm depths (Table 1). In the 0–15 cm depth, soil pH was highest in limited-irrigation cropping systems (LINTF and LINTC), which was not significantly higher than NG but higher than dryland cropping systems (DLCTF and DLNTF). In the 15–30 cm depth, limited-irrigation cropping systems had significantly higher soil pH (7.9–8.0) than dryland systems and NG (7.3–7.6).

Soil EC was significantly lower in dryland cropping systems (0.11–0.12 ds m^−1^) than in limited-irrigation systems and NG (0.20–0.22 ds m^−1^) in the 0–15 cm depth. In the 15–30 cm depth, the EC was significantly higher in DLCTF and LINTF than in NG and DLNTF (Table 1). Soil EC in LINTC was intermediate between other treatments.

Soil CEC did not differ between treatments in either depth. Soil CEC was in the range of 15.9–22.7 cmol_c_ kg^−1^ in the 0–15 cm depth and 19.0–23.9 cmol _c_ kg^−1^ in the 15–30 cm depth. Similarly, soil Db was in the range of 1.06–1.25 g cm^−3^ in the 0–15 cm and 1.14–1.30 g cm^−3^ in the 15–30 cm depth and no significant difference between treatments in either depth.

**Table 1:** Soil properties in 0–15 cm and 15–30 cm soil depths under various cropping systems and native grassland.

Soil depth (cm)	Treatment	Soil pH	Electrical conductivity (ds m^−1^)	CEC (cmol_c_ kg^−1^)	Bulk density (g cm^−3^)
0–15	DLCTF^a^	7.40 ± 0.04b	0.12 ± 0.13b	16.5 ± 0.43	1.06 ± 0.02
	DLNTF	6.90 ± 0.04c^b^	0.11 ± 0.03b	15.9 ± 0.26	1.18 ± 0.02
	LINTF	7.86+0.04a	0.22 ± 0.03a	19.7 ± 0.34	1.21 ± 0.01
	LINTC	7.83 ± 0.11a	0.21 ± 0.17a	22.7 ± 1.32	1.25 ± 0.12
	NG	7.56 ± 0.08ab	0.20 ± 0.11a	20.1 ± 0.24	1.14 ± 0.08
15–30	DLCTF	7.33 ± 0.02c	0.49 ± 0.19a	20.0 ± 0.23	1.30 ± 0.04
	DLNTF	7.40 ± 0.07c	0.19 ± 0.07c	19.3 ± 0.50	1.29 ± 0.07
	LINTF	7.90 ± 0.00a	0.42 ± 0.22ab	23.4 ± 0.72	1.24 ± 0.28
	LINTC	8.00 ± 0.04a	0.25 ± 0.07bc	23.9 ± 1.02	1.14 ± 0.08
	NG	7.63 ± 0.02b	0.13 ± 0.06c	19.0 ± 0.37	1.21 ± 0.58

**Notes.**

a DLCTF, dryland winter wheat-sorghum-fallow with conventional tillage; DLNTF, dryland winter wheat-sorghum-fallow with no-tillage; LINTF, limited-irrigation winter wheat-sorghum-fallow with no-tillage; LINTC, limited-irrigation winter wheat-sorghum-fallow with no-tillage and cover crops; NG, native grassland; and CEC, cation exchange capacity.

b Mean values (±standard error) followed by different lowercase letters in a column indicate a significant difference between treatments (*p* ≤ 0.05).

**Figure 1:**
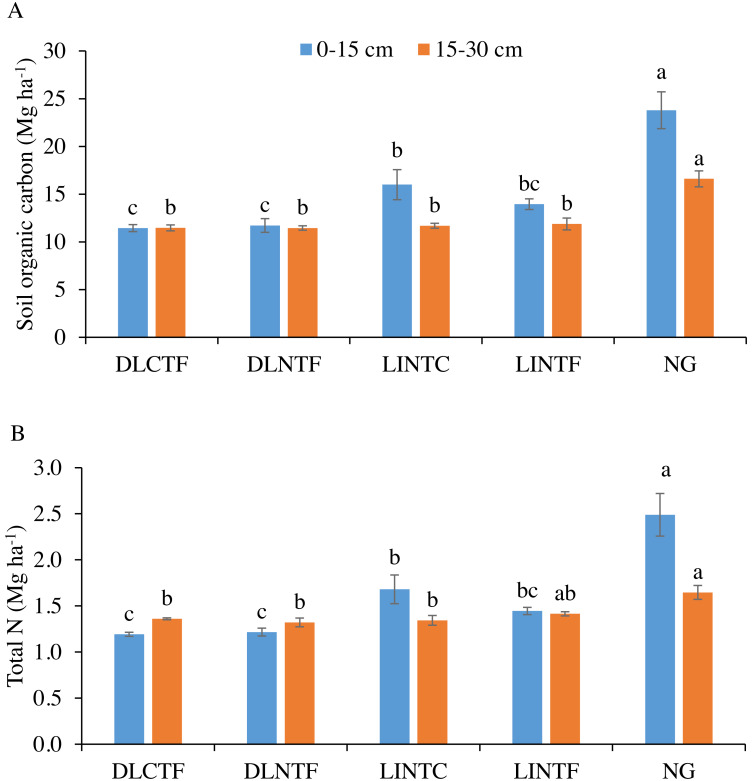
Soil organic carbon (A) and total N (B) in 0–15 cm and 15–30 cm soil depths under various cropping systems and native grassland. DLCTF, dryland winter wheat–sorghum–fallow with conventional tillage; DLNTF, dryland winter wheat–sorghum–fallow with no-tillage; LINTC, limited-irrigation winter wheat–sorghum–fallow with no-tillage and cover crops; LINTF, limited-irrigation winter wheat–sorghum–fallow with no-tillage, and NG = native grassland. Mean values (±standard error) followed by different lowercase letters indicate a significant difference between treatments within a soil depth (*p* ≤ 0.05).

The SOC content was significantly greater in NG than all cropping systems in both soil depths. In the 0–15 cm depth of cropland systems, LINTC had significantly greater SOC than dryland cropping systems (Fig. 1A). The SOC in LINTF was intermediate between dryland cropping systems and LINTC. In the 15–30 cm depth, there was no difference in SOC content between irrigated and dryland cropping systems. The SOC in the 0–30 cm depth was 14% less in dryland systems, while it was 51% more in grasslands than limited-irrigation systems.

Soil total N content followed a similar trend as SOC that NG had significantly greater total N than all cropping systems in both depths. Total N in the 0–15 cm depth of LINTC was significantly greater than dryland systems but not significantly greater than LINTF (Fig. 1B). The total N in the 15–30 cm depth was greater in NG than in dryland cropping systems and LINTC. In LINTF, total N was intermediate between NG and other systems. Total N content was 13% less in the 0–30 cm depth of dryland and 41% more in the same depth of NG compared to limited-irrigation cropping systems.

**Figure 2:**
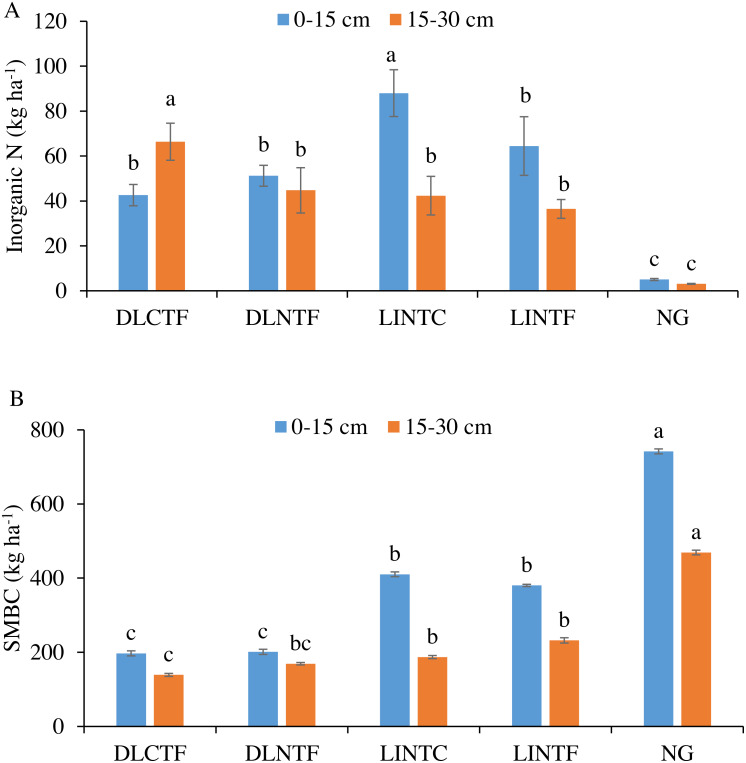
Soil inorganic N (A) and microbial biomass carbon (SMBC) (B) in 0–15 cm and 15–30 cm soil depths under various agroecosystems. DLCTF, dryland winter wheat–sorghum–fallow with conventional tillage; DLNTF, dryland winter wheat–sorghum–fallow with no-tillage; LINTC, limited-irrigation winter wheat–sorghum–fallow with no-tillage and cover crops; LINTF, limited-irrigation winter wheat–sorghum–fallow with no-tillage; and NG, native grassland. Mean values (±standard error) followed by different lowercase letters indicate a significant difference between treatments within a soil depth (*p* ≤ 0.05).

Soil inorganic N in the 0–15 cm depth was greater in LINTC than other cropping systems (Fig. 2A). The inorganic N was considerably lower in NG than all cropping systems. In the 15–30 cm, it was significantly greater in DLCTF than all other systems. The inorganic N in limited-irrigation cropping systems and DLNTF was significantly lower than DLCTF but greater than NG.

The SMBC was significantly greater in NG than all the cropping systems in both soil depths (Fig. 2B). The SMBC in the 0–15 cm depth was highest in NG, followed by limited-irrigation and dryland cropping systems. The SMBC in the 15–30 cm depth followed the trend of 0–15 cm depth, with no significant difference between DLNTF and DLCTF, as well as irrigated systems.

**Figure 3:**
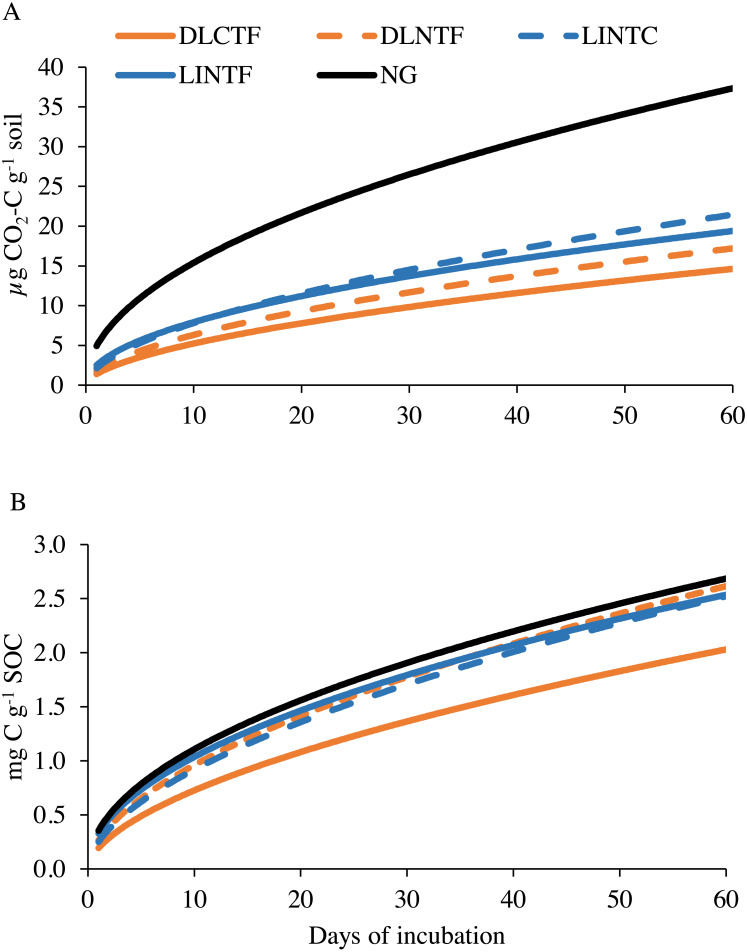
Modeled soil CO2-C emissions on a per-unit soil (A) and per-unit soil organic carbon content (B) basis in various cropping systems and native grassland. DLCTF, dryland winter wheat–sorghum–fallow with conventional tillage; DLNTF, dryland winter wheat–sorghum–fallow with no-tillage; LINTC, limited-irrigation winter wheat–sorghum–fallow with no-tillage and cover crops; LINTF, limited-irrigation winter wheat–sorghum–fallow with no-tillage; and NG, native grassland.

**Figure 4:**
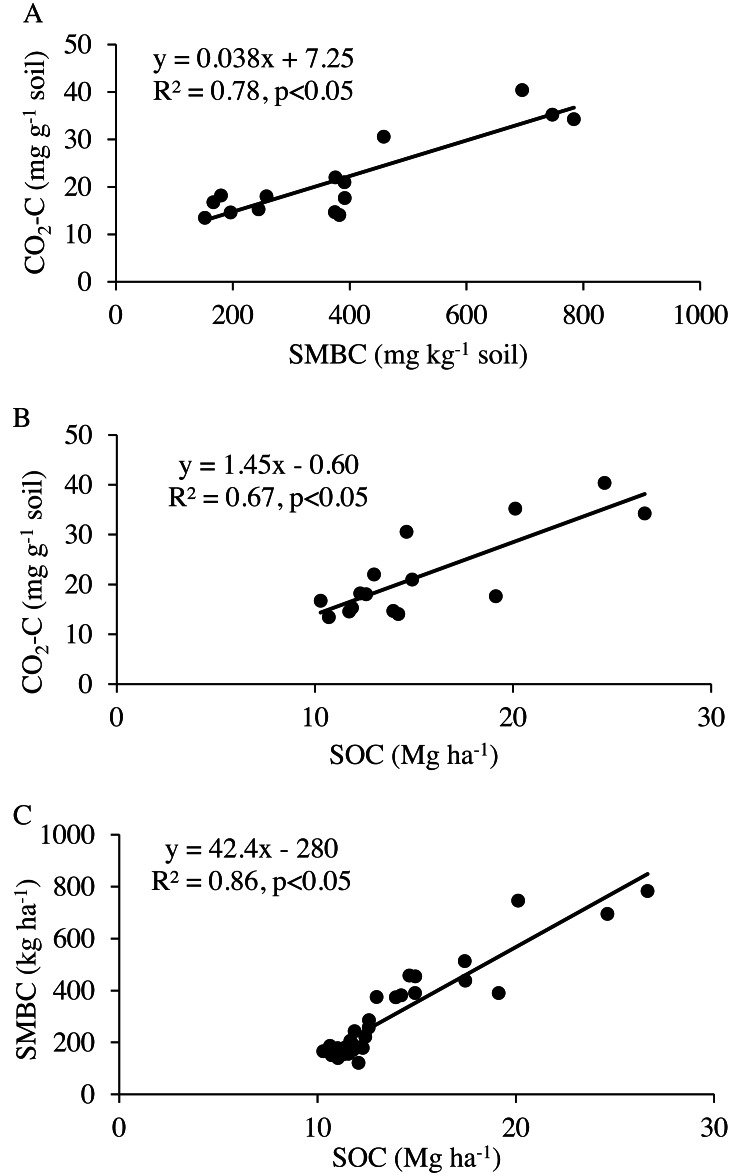
Regression analysis between (A) total CO_2_-C release during eight weeks of incubation and microbial biomass carbon (SMBC), (B) soil organic carbon (SOC), and (C) between SMBC and SOC contents.

The microbial respiration measured as CO_2_-C release during laboratory incubation varied among agroecosystems. The NG released the highest amount of CO_2_-C, followed by limited-irrigation cropping systems and dryland cropping systems (Fig. 3A). Since these agroecosystems varied in SOC content, we reanalyzed the data by normalizing with SOC content in the system. The CO_2_-C release was consistent among NG, limited-irrigation cropping systems, and DLNTF (Fig. 3B). Only DLCTF had a lower microbial respiration than other treatments. The first-order kinetic model showed *k* in the range of 0.49–0.57, while labile substrate (*C*_0_) was in the range of 1.41–5.1 mg kg^−1^ soil (0.22–0.38 g kg^−1^ SOC), indicating most of the variation in microbial respiration was related to labile substrate present in the soil. The regression analysis showed that microbial respiration was strongly related to the SOC content and microbial biomass (Fig. 4). The model fit-statistics (r, RMSE, NRMSE) revealed that the model explained well the variability in CO_2_-C release from these systems (Table 2).

**Table 2:** Model-fit statistics for CO_2_-C and N_2_O-N emissions data with and without normalizing to soil organic carbon and total nitrogen contents.

Condition	Treatment	CO_2_-C					N_2_O-N				
		*C*_0_	*K*	R	RMSE	NRMSE	*N*_0_	*K*	r	RMSE	NRMSE
Before normalizing to SOC/TN	DLCTF	1.41	0.57	0.998	0.295	3.93	1.12	0.55	0.994	0.404	5.45
	DLNTF	1.75	0.55	0.997	0.513	5.69	0.85	0.60	0.994	0.399	5.97
	LINTC	2.13	0.56	0.999	0.340	3.06	1.65	0.52	0.983	0.864	8.87
	LINTF	2.51	0.50	0.999	0.262	2.46	2.24	0.47	0.995	0.487	4.35
	NG	5.01	0.49	0.998	0.607	2.95	1.30	0.53	0.995	0.441	5.58
After normalizing to SOC/TN	DLCTF	0.22	0.57	0.998	0.047	4.02	1.67	0.55	0.987	0.598	5.40
	DLNTF	0.26	0.56	0.997	0.062	4.69	1.19	0.60	0.983	0.550	5.85
	LINTC	0.25	0.56	0.999	0.041	3.10	1.85	0.52	0.985	0.971	8.91
	LINTF	0.28	0.50	0.999	0.029	2.45	3.85	0.47	0.975	0.845	4.40
	NG	0.38	0.49	0.998	0.045	2.88	0.95	0.53	0.975	0.313	5.40

**Notes.**

a DLCTF, dryland winter wheatsorghumfallow with conventional tillage, DLNTF, dryland winter wheatsorghumfallow with no-tillage, LINTF, limited-irrigation winter wheatsorghumfallow with no-tillage, LINTC, limited-irrigation winter wheatsorghumfallow with no-tillage and cover crops, NG, native grassland.

**Figure 5:**
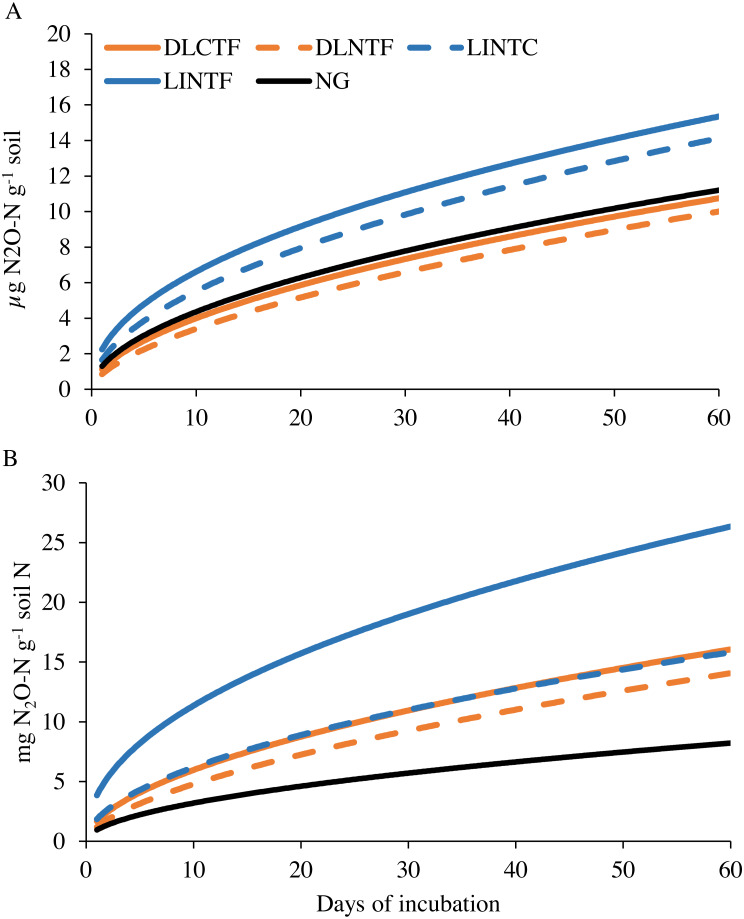
Modeled soil N_2_O-N emissions on a per-unit soil (A) and total N content (B) basis in various cropping systems and native grassland. DLCTF, dryland winter wheat–sorghum–fallow with conventional tillage; DLNTF, dryland winter wheat–sorghum–fallow with no-tillage; LINTC, limited-irrigation winter wheat–sorghum–fallow with no-tillage and cover crops; LINTF, limited-irrigation winter wheat–sorghum–fallow with no-tillage; and NG, native grassland.

The N_2_O-N release during laboratory incubation showed a higher rate of N release from limited-irrigation cropping systems than dryland cropping systems and NG (Fig. 5A). When the N_2_O-N release was normalized to total N, the value was even smaller per unit N in NG than all the cropping systems (Fig. 5B). The N mineralization constant *k* was in the range of 0.47–0.60, and *N*_0_ was in the range of 0.85–2.24 mg kg^−1^ soil (0.95–3.85 g kg^−1^ TN), showing the broader range in N_2_O-N release relative to CO_2_-C release (Table 2). The model-fit statistics (r, RMSE, NRMSE) for observed versus modeled N_2_O-N release revealed that the first-order kinetic model explained well the variability in N_2_O-N release from these systems. Soil N_2_O-N release followed a weak trend with inorganic N (*p* = 0.12), and it was not related to the total N content.

## Discussion

This study demonstrated an increase in SOC and N with native grassland restoration in agroecosystems transitioning to dryland, with greater magnitude of increase in biologically active SOC and N. Grasslands had 100% more SMBC and 51% more SOC than irrigated cropping systems and 243% more SMBC and 66% more SOC than dryland cropping systems. Biologically active soil organic matter is derived from high plant biomass production along with deeper and denser root systems of perennial grasses than annual crops (DuPont et al., 2010; Guo & Gifford , (2002). The SOC under perennial grasses also increases from a higher root: shoot ratio and low decomposition rate of roots from perennial grasses than from annual crops (Yang et al., 2019). Perennial grasses also increase microbial biomass, thereby contributing to more SOC and N accumulation in grasslands compared to croplands (Ghimire et al., 2019). Our previous study shows two- to six-times greater biomass C inputs in grassland than in cropland soils (Ghimire, Machado & Rhinhart, 2015). In the current study, native grasslands accumulated more C in the soil profile with a net increase in SOC sequestration. The SOC in the NG was even greater than the cover crop-integrated cropping system (LINTC). Grassland treatment was in place for 14 years, while the cover cropping treatment was in place for only three years.

The SOC sequestration or loss in agricultural soils depends on management practices, biomass inputs, and quality (Lehmann & Kleber, 2015; Ghimire, Machado & Bista, 2019). Above- and belowground plant production decreases with a decrease in water availability for irrigation. Reduced C and nutrients inputs from plant biomass recycling and enhanced microbial respiration with repeated disturbance can deplete SOC and nutrients in cropping systems transitioning to dryland. We did not monitor plant biomass production in different cropping systems and grasslands in this study. However, a significantly lower SOC, total and inorganic N, and SMBC in drylands than irrigated cropping systems demonstrated the effects of this transition. The SMBC and SOC were 42% and 14% lower in dryland than in irrigated cropping systems, irrespective of tillage management or cover cropping. The rate of CO_2_-C and N_2_O-N release per unit SOC and N, respectively, were similar across all agroecosystems compared in this study, which suggests no change in the rate of SOC and N loss despite the difference in inputs during the transition to dryland production. Agroecosystems in the semiarid regions continuously change SOC and nutrient status until they reach a new equilibrium (Bista et al., 2016). It appears SOC in dryland cropping systems in this study will continue to decline until it reaches a lower equilibrium.

Adoption of no-tillage typically increases SOC in surface soil (Mitchell et al., 2015; Sainju, Jabro & Stevens, 2008). Three years of no-tillage after 11 years of dryland cropping and conventional tillage was not enough to detect increase in SOC in this hot, dry semiarid agroecosystem. Management systems that reduce soil disturbance along with cropping system intensification could increase SOC and maintain net positive ecosystem carbon balance compared to no-tillage alone in hot, dry environments of the Southern High Plains (Rosenzweig, Stromberger & Schipanski, 2018; Thapa et al., 2019). Cropping system intensification increases SOC through greater C inputs to soil, as well as through increased fungal biomass and soil aggregation (Rosenzweig, Stromberger & Schipanski, 2018). Our study did not compare crop intensification and diversification options in drylands, but cover crop-integrated no-tillage system (LINTC) had numerically higher SOC than without cover crops under limited irrigation. A greater magnitude of increase in biologically active organic matter was observed with cover cropping than without, which suggests a potential long-term improvement in soil health through soil C accumulation. Cover crop plots in this study used a mixture of legume, brassica, and grass species. The legume and brassica cover crops produce high-quality biomass due to their low carbon to nitrogen ratios (Finney, White & Kaye , 2016). A mixture of cover crops also diversify microbial substrates through diverse root exudates and aboveground plant parts, which alters decomposition dynamics of crop residue inputs, leading to improvements in soil nutrient cycling and SOC accumulation (Mc Daniel et al., 2016). Our study shows higher SMBC and microbial respiration during eight weeks of incubation with cover crops than without cover crops suggesting the possibility of improving SOC in long-term.

The first-order kinetic model was used to evaluate the labile SOC and N pools and decomposition kinetics under various management systems. The decomposition rate constant (*K*) was highest in conventionally tilled drylands, followed by limited-irrigation croplands, and native grasslands. It is not surprising that decomposition rate increases with increasing disturbance and annual cropping compared to perennial grasses. Previous studies show a positive relationship between labile SOC pools, CO_2_-C production, and SOC sequestration (Ahlering, Fargione & Parton, 2016; Ghimire et al., 2019; Ghimire, Machado & Bista, 2019; Six, Elliott & Paustian, 2000). However, we expected a lower decomposition rate in dryland cropping systems because of low biomass recycling in dryland cropping systems. The CO_2_-C release did not vary between agroecosystems when normalized to SOC content, except in DLCTF. Relatively small CO_2_-C fluxes in DLCTF when normalized to SOC content may be due to loss of labile carbon during repeated tillage activities.

The release of N_2_O-N was highest in limited-irrigation cropping systems, followed by native grassland and dryland cropping systems. The highest rate of N release in limited-irrigation systems may have been due to high N input through external sources (fertilizer and atmospheric N fixation). Limited-irrigation systems received more N (97 kg N ha^−1^ for sorghum) than dryland systems (33.6 kg N ha^−1^ for sorghum). Although the relationship was not statistically significant for both N pools (*p* ≤** 0.05), there was a better correlation with inorganic N than with total N. The lowest N_2_O-N release and inorganic N despite highest total N storage in native grassland suggests N limit the grassland productivity. Grasslands are often low in soil inorganic N because they do not receive any external N inputs and solely depend on internal N cycling. High C inputs for several years from biomass recycling along with no external N inputs increased SOC in grasslands, but low soil inorganic N and N_2_O-N release. This shows possibility of mitigating global warming through grassland restoration, considering 310 times higher global warming potential of N_2_O than that of CO_2_ (IPCC, 2007). Evaluating CO_2_ and N_2_O emissions under grasslands and croplands in the Northern High Plains, Ahlering, Fargione & Parton (2016) reported the potential to offset SOC depletion and contribution to climate mitigation by avoiding conversion of grasslands into agricultural lands.

Water level in the Ogallala Aquifer has been depleting and the decrease in SOC and N levels are underway (Cano et al., 2018). Areas currently under limited-irrigation crop production could benefit from cover cropping in long-term because we observed small but positive effects of cover crops on SOC and N storage within three years of study establishment. However, about 24% of currently irrigated acreage is projected to be converted to dryland by 2100 and 13% of these areas are not suitable for dryland crop production (Deines et al., 2020). In such areas, grassland establishment could restore SOC and N in the soil profile. There was a strong positive relationship between microbial respiration, SMBC, and SOC. Specifically, the response of labile SOC components, including SMBC and microbial respiration were very clear. Agroecosystems that reduce soil disturbance, integrate cover crops, and increase the residue inputs can increase SOC and N storage and improve the sustainability of agriculture in semiarid regions transitioning from limited-irrigation to dryland management.

## Conclusions

A vast area of the High Plains of USA is facing challenges in maintaining irrigated crop production due to a continuous depletion of the water level in the Ogallala Aquifer. Management selection plays a crucial role on SOC and N cycling in semiarid agroecosystems transiting to dryland. Our study demonstrated loss of SOC and N in agroecosystems transitioned to dryland crop-fallow systems and greater magnitude of change was observed in the biologically active fraction of soil organic matter. Grassland restoration could sequester C and nutrients in the soil profile. Although no-tillage cover crops were planted for only three years, there was a small increase in SOC components. The large CO_2_ fluxes from soils with higher SOC agroecosystem suggests a critical role of residue inputs in C cycling. The N cycling was more related to the external N input via fertilizer application. An in-depth and regional-scale study will reveal the role of various agroecological measures in mitigating SOC and N loss during transition to dryland production. This study demonstrated that no-tillage and cover cropping had a small but positive change in SOC storage and nutrient cycling. Grassland restoration can increase SOC and N storage and improve agroecosystem sustainability in the hot, dry, semiarid agroecosystems facing transition to dryland.

##  Supplemental Information

10.7717/peerj.10199/supp-1 Supplemental Information 1 Management selection determines soil organic matter dynamics during transition to dryland in semiarid agroecosystems

10.7717/peerj.10199/supp-2 Supplemental Information 2 Raw data for Figs. 1–5 DLCTF = dryland winter wheat–sorghum–fallow with conventional tillage, DLNTF = dryland winter wheat–sorghum–fallow with no-tillage, LINTC, limited-irrigation winter wheat–sorghum–fallow with no-tillage and cover crops, LINTF, limited-irrigation winter wheat–sorghum–fallow with no-tillage, and NG = native grassland.

## Additional Information and Declarations

### Data Availability

The following information was supplied regarding data availability: Raw data are available in the Supplemental Files.
